# The effect of an improved ICU physical environment on outcomes and post-ICU recovery—a protocol

**DOI:** 10.1186/s13063-024-08222-6

**Published:** 2024-06-11

**Authors:** Oystein Tronstad, Barbara Zangerl, Sue Patterson, Dylan Flaws, Stephanie Yerkovich, Irene Szollosi, Nicole White, Veronica Garcia-Hansen, Francisca Rodriguez Leonard, Benjamin D. Weger, Frédéric Gachon, David Brain, Jayshree Lavana, Carol Hodgson, John F. Fraser

**Affiliations:** 1 Critical Care Research Group, The Prince Charles Hospital, Level 3 Clinical Sciences Building, Rode Road, Chermside, Brisbane, Qld 4032 Australia; 2 Faculty of Medicine, University of Queensland, Brisbane, Australia; 3 Physiotherapy Department, The Prince Charles Hospital, Brisbane, Australia; 4 School of Dentistry, University of Queensland, Brisbane, Australia; 5 Department of Mental Health, Metro North Mental Health, Caboolture Hospital, Caboolture, Australia; 6 School of Clinical Sciences, Queensland University of Technology, Brisbane, Australia; 7 Menzies School of Health Research and Faculty of Health, Queensland University of Technology, Brisbane, Australia; 8 Sleep Disorders Centre, The Prince Charles Hospital, Brisbane, Australia; 9 Australian Centre for Health Services Innovation, School of Public Health and Social Work, Queensland University of Technology, Brisbane, Australia; 10 School of Architecture and Built Environment, Faculty of Engineering, Queensland University of Technology, Brisbane, Australia; 11 Institute for Molecular Bioscience, The University of Queensland, Brisbane, Australia; 12 Adult Intensive Care Services, The Prince Charles Hospital, Brisbane, QLD Australia; 13 Australian and New Zealand Intensive Care Research Centre, School of Public Health and Preventive Medicine, Monash University, Melbourne, Australia; 14 Division of Clinical Trial and Cohort Studies, School of Public Health and Preventive Medicine, Monash University, Melbourne, Australia; 15 Physiotherapy Department, Alfred Hospital, Melbourne, Australia

**Keywords:** Co-design, Environment, ICU, Light, Noise, Study protocol

## Abstract

**Background:**

Intensive care medicine continues to improve, with advances in technology and care provision leading to improved patient survival. However, this has not been matched by similar advances in ICU bedspace design. Environmental factors including excessive noise, suboptimal lighting, and lack of natural lights and views can adversely impact staff wellbeing and short- and long-term patient outcomes. The personal, social, and economic costs associated with this are potentially large. The ICU of the Future project was conceived to address these issues. This is a mixed-method project, aiming to improve the ICU bedspace environment and assess impact on patient outcomes. Two innovative and adaptive ICU bedspaces capable of being individualised to patients’ personal and changing needs were co-designed and implemented. The aim of this study is to evaluate the effect of an improved ICU bedspace environment on patient outcomes and operational impact.

**Methods:**

This is a prospective multi-component, mixed methods study including a randomised controlled trial. Over a 2-year study period, the two upgraded bedspaces will serve as intervention beds, while the remaining 25 bedspaces in the study ICU function as control beds. Study components encompass (1) an objective environmental assessment; (2) a qualitative investigation of the ICU environment and its impact from the perspective of patients, families, and staff; (3) sleep investigations; (4) circadian rhythm investigations; (5) delirium measurements; (6) assessment of medium-term patient outcomes; and (7) a health economic evaluation.

**Discussion:**

Despite growing evidence of the negative impact the ICU environment can have on patient recovery, this is an area of critical care medicine that is understudied and commonly not considered when ICUs are being designed. This study will provide new information on how an improved ICU environment impact holistic patient recovery and outcomes, potentially influencing ICU design worldwide.

**Trial registration:**

ACTRN12623000541606. Registered on May 22, 2023. https://www.anzctr.org.au/Trial/Registration/TrialReview.aspx?id=385845&isReview=true.

**Supplementary Information:**

The online version contains supplementary material available at 10.1186/s13063-024-08222-6.

## Background

Globally, intensive care units (ICUs) provide critical care and life support to 13–20 million acutely ill and injured patients every year [1]. Technological and clinical advances have contributed to increased survival of patients in recent years. These advances, however, have not been matched by advances in the design of ICUs. Despite longstanding recognition of the interrelationship between the environment and health, contemporary ICUs are increasingly medicalised, busy, and noisy environments that is suggested to hinder rather than promote recovery [2–4].

There is increasing evidence that the ICU bedspace environment, including excessive sound and alarms, artificial lighting, and lack of access to natural light and views, has negative effects on patients, their family members, and staff [5–9]. However, existing evidence is limited, and there is therefore inadequate, if any, evidence available to demonstrate causation or the ability to discern whether these negative outcomes are simply effects of critical illness and hospitalisation alone, as previous studies have not compared different or upgraded ICU bedspaces and impact on outcomes. These environmental and design factors contribute to the sleep deprivation and delirium commonly experienced by patients admitted to ICU, thereby potentially contributing towards increasing morbidity and mortality [10–17]. Environmental factors such as excessive sound or noise may adversely affect staff health and performance [18, 19]. The negative impact on staff wellbeing and mental health may therefore contribute to the high nursing turnover, reported to be as high as 36% annually, and thereby on current nursing shortages [18–22]. The cost of hospital employee turnover is estimated to be more than 5% of hospital budgets; replacing an experienced ICU nurse can cost up to USD $100,000 [22, 23].

The impact on patients may extend beyond admission, with disrupted sleep and delirium contributing towards the development of post-intensive care syndrome (PICS), a collection of complications including cognitive, physical, mental, and psychological disability, which affects up to 80% of patients discharged from critical care [24, 25]. The ongoing health problems and disability associated with PICS can severely impact the quality of life of patients and their families [26, 27]. Many ICU survivors never return to work and require significant caregiver support. This may detrimentally impact on their family members’ ability to work and their earning capacity, leaving them unable to provide for the family financially, with close to one third of patients reporting losing most or all family savings and their major source of income after serious illness [28]. Associated personal, social, and economic costs are immense.

To redress these issues, investment should be targeted towards solving these widely recognised design problems and evaluating the outcomes of environmental upgrades. Previous studies have suggested that a modified ICU environment is associated with a reduced delirium rate and ICU length of stay [29]. However, to date, there are limited studies directly investigating the relationship between ICU bedspace environmental upgrades and patient outcomes [30].

The ICU of the Future project is a mixed-method project, aiming to improve the ICU bedspace environment and assess impact on patient outcomes. This project commenced with modifiable environmental, technological, and design features contributing to suboptimal outcomes being identified via qualitative patient, family, and staff interviews [4, 31] and quantitative studies [32]. This was followed by a co-design process, used to redesign and develop an innovative and adaptive ICU bedspace capable of being individualised to patients’ personal and changing needs and aimed to optimise clinical efficiency, patient experience, and outcomes [33]. The new bedspace design has been implemented in the study ICU with patients admitted from January 2023. Table 1 summarises the main changes to the implemented bedspaces.

**Table 1:** Main changes implemented for the upgraded bedspaces

Environmental factor addressed	What was implemented
Sound/noise reduction	
1. Improved sound absorption	1. Acoustically absorbent ceiling tiles and walls (satisfying infection control criteria) and softer floor vinyl
2. Improved sound blocking	2. Added doors to open-plan bedspaces with double layer of extra thick acoustic glass and optimal seals
3. Reduced sound production within bedspace	3. Repositioned alarms away from patients’ head, reconfigured and reduced alarm numbers, staff education
4. Improved sound control within bedspace	4. Introduced sound masking and beds with speakers built into them with the ability to link wirelessly to a patient entertainment system, enabling, e.g. individualised music therapy
Light	
1. Optimised lighting specific for two internal and windowless bedspaces	1. Installed a timed and programmed circadian lighting solution able to be modified to patients’ specific needs
Patient connectivity, stimulation, distraction, and engagement	
1. Improved patient stimulation and distraction	1. Patient entertainment system, materials, and colours were carefully chosen to make the space feel less clinical and overwhelming
2. Improved views and connectivity with nature in windowless bedspaces	2. Virtual window and artificial skylight with several available videos that can be selected based on personal preferences
3. Improved connectivity with family and friends	3. Virtual visiting
Staff solutions	
1. Support communication and workflow	1. Updated nurse call system installed, mobile solutions replaced static ones where able (e.g. workstation on wheels), decluttering the bedspaces where able

The aim of the study described in this protocol is to evaluate the impact of the ICU bedspace environmental upgrade on short- and medium-term patient outcomes and operational impact (health economic evaluation, objective environmental evaluation, and staff experience). Findings of the project will be relevant to a range of stakeholders, including health care administrators and providers, clinicians, patients, and their families.

## Design and methods

### Setting

This study will be conducted in a 27-bed adult ICU at a large urban Australian tertiary referral hospital specialising in cardiothoracic surgery and medicine. The ICU originally comprised 21 open-plan bedspaces and six isolation beds in three ‘pods’ of nine beds each, surrounding a central nursing station. Two of the windowless open-plan bedspaces were modified and will serve as the intervention beds; the remaining 25 will function as control beds. The unit admits approximately 1800 patients annually.

### Study design and plan

**Fig. 1:**
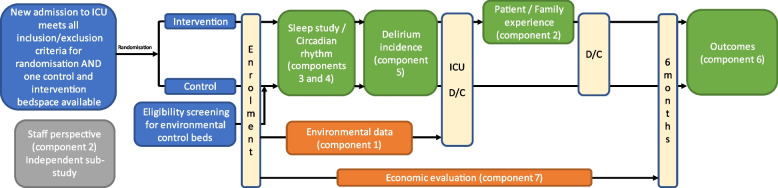
Randomisation, recruitment, enrolment, and study plan

This is a prospective multi-component, mixed methods study including a randomised controlled trial (RCT) (Fig. 1). The study followed the SPIRIT reporting guidelines (Supplementary File) [34].

Study components encompass:

1. An objective environmental assessment
2. A description of the ICU environment from the perspective of patients, families, and staff
3. Sleep and circadian rhythm investigations
4. Delirium measurements
5. Assessment of medium-term patient outcomes 6 months after discharge from ICU
6. A health economic evaluation

Data pertinent to each component will be collected and analysed using methods appropriate to type and source of data and analysed separately before being synthesised in a summative assessment.

#### RCT component

The primary study assessing patient outcomes (study components 3–6 described below) is an RCT, in which the two upgraded bedspaces are defined as intervention, while the 25 ‘conventional’ bedspaces serve as control. Eligibility screening, consent, and randomised allocation is a two-step process. All patients admitted to ICU are eligible to be randomised 1:1, except those deemed unsuitable for admission to the intervention bedspaces:

- Patients requiring extracorporeal membrane oxygenation, intra-aortic balloon pump, or dialysis
- Patients admitted to ICU immediately following cardiac surgery, including transplantation

Patients will be randomly assigned to bedspaces as follows:

1. A random sequence of allocations to intervention and control bedspaces was established by the research coordinator and sealed in numbered, opaque envelopes. ICU floor coordinators and clinical personnel are blinded to this sequence until revealed at randomisation.
2. Randomisation is completed by the ICU floor coordinator/admitting ICU doctor when:
  At least one intervention and one control bedspace are available at the time a patient is accepted for admission.
  The patient is eligible for randomisation (see above).
3. The ICU floor coordinator or responsible clinician on duty opens the next envelope in sequence to reveal allocation of the patient to an intervention or control bedspace.
4. The patient is assigned to the allocated bed.
5. Patients will then be assessed for eligibility for participation in the study by a member of the research team (see ‘Recruitment’ section below).

#### Case–control sub-study

Bedspaces in the study ICU differ regarding access to windows and natural light, potentially confounding outcomes. To investigate the effect of these variables, this study involves a concurrent observational case–control study utilising the three bedspaces that are most similar to the intervention bedspaces. These three internal and windowless bedspaces will function as ‘environmental control’ bedspaces. Thus, all patients admitted to these three bedspaces will also be invited to participate using the same protocols as the main RCT, but analysis will be adjusted to account for any potential bias in admission diagnosis, severity of illness, or other baseline data compared to the RCT cohort. Where patients are allocated to the environmental control bedspaces as part of the RCT, no further action will be taken, but patient data will also be utilised for the independent sub-analysis comparing intervention to environmental control bedspaces. Additionally, patients admitted to the intervention beds without being randomised will be approached for participation in the study as case control patients.

### Recruitment

For an estimated study period of 2 years, all patients randomised to an intervention or control ICU bedspace as part of the RCT or admitted to environmental control bedspaces as part of the case–control study will be screened for suitability to participate in the research study. Suitable patients will be approached by a member of the research team as soon as possible after admission to ICU, in consultation with the treating clinical team. As patient status commonly changes throughout ICU admission, patients may be screened daily until discharge and may be recruited to the study at any time during their admission to ICU if suitable based on the following inclusion/exclusion criteria:

#### Inclusion criteria

- Patient residing in Australia
- Patients sufficiently fluent in English to complete recruitment and data collection processes
- Patients expected to remain in ICU for > 24 h

#### Exclusion criteria

- Patient or legal representative unable or unwilling to provide consent.
- Patients less than 18 years of age.
- Death is deemed imminent.
- Patient deeply or moderately sedated (RASS score ≤  − 3).
- Recent substantial neurological insult (e.g. stroke).
- Patient deemed agitated, aggressive, or displays unpredictable behaviour.

**Fig. 2:**
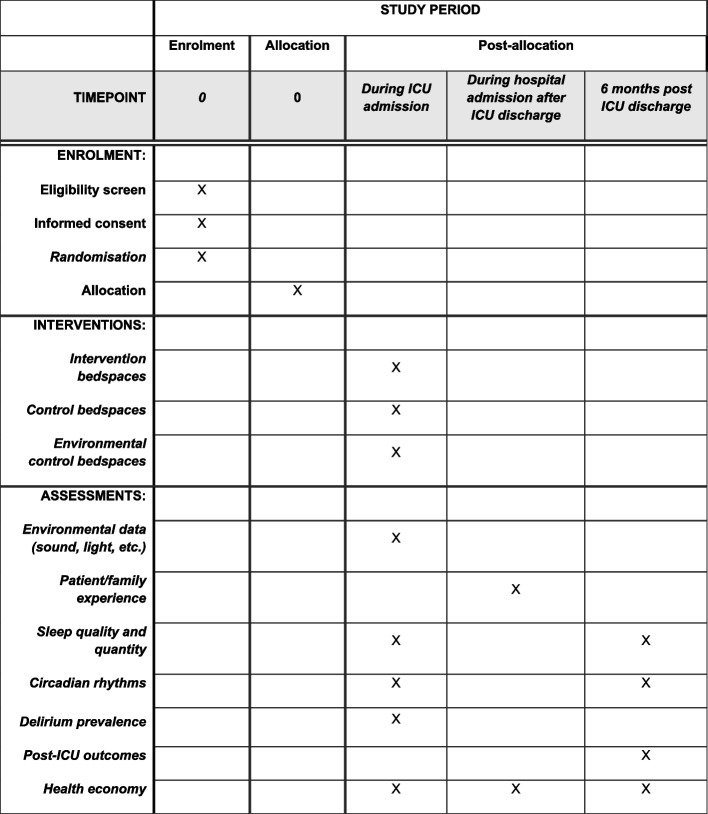
Study schedule of enrolment, intervention, and assessments

Patients will be informed of the purpose and aims of the study and informed consent will be obtained prior to being enrolled in the study. If patients are unable to consent, the substitute decision-maker or next-of-kin will be approached to consider participation. Figure 2 summarises the enrolment, intervention, and assessment timeline.

Patients for whom consent is obtained will complete the study components listed below. Based on annual admission numbers and current admission diagnoses and presentations, the average number of patients eligible for randomisation per bedspace in the study facility is approximately 30 per year. We therefore estimate that approximately 250 patients will be randomised over 2 years. We expect that approximately 50 patients will be ineligible for participation for various reasons and based on other studies in the unit we have conservatively estimated a 50% patient decline rate. We therefore estimate that approximately 100 patients will provide consent and be recruited to this study over the 2-year study period.

A power calculation for this study is not possible, due to the lack of studies reporting the impact of environmental upgrades on patient outcomes. Therefore, an interim analysis will be conducted after 6 months of data collection, focussing on recruitment rate, completion rate for the various study components, and in-hospital outcome measures, with a power calculation performed based on this data to determine the number of participants required.

### Data collection and monitoring, study components, and data analysis

Data describing participant demographics and ICU admission will be collected from hospital records and recorded on case report forms (CRFs). Data collected will include date of birth, gender, hospital and ICU length of stay, ICU and hospital survival, ICU readmission rates, mechanical ventilation time, details of their illness, medical treatment, and existing comorbidities. Completed CRFs will be entered into an electronic database. Patient characteristics and ICU admission data will be described using summary statistics. The number of patients approached and consented will be charted, together with the number who dropped out or died.

A data monitoring committee was not considered as this was a low-risk intervention. Data monitoring and study conduct is reviewed 6 monthly by the research investigators and submitted for ethics committee review yearly.

In addition to data from the study components being individually analysed and reported as described below, data analysis will also investigate the relationships between different outcomes/components. Inter-related components that will be explored include whether observed differences in sleep architecture is correlated with changes in delirium outcomes, and whether a difference in the maintenance of circadian rhythm between intervention and control bedspaces impacts the incidence of PICS at 6-month post-ICU discharge.

#### Component 1: objective environmental assessment of the upgraded ICU environment

This observational component of the study has been designed to generate a comprehensive quantitative account of the upgraded ICU environment with reference to physical space, sound, acoustics, lighting, temperature, and humidity, and compare with previously collected baseline/control data. The intervention bedspaces will be continuously monitored for the study period, with sound levels (dB/dBA), light levels (lux), temperature (°C), and humidity (%) all being monitored via wireless integrated environmental monitoring sensors (Senseagent LP4-204 Hilbert Sensor). These sensors communicate via a wireless LP4-391 Neumann Gateway, with members of the research team able to download the data from a cloud-based database into an Excel spreadsheet. The evaluation of sound levels will also include audits of the number and type of alarms, with alarm data downloaded from key equipment in ICU, including patient monitors and ventilators.

A detailed acoustic and lighting evaluation will be performed. A specialist acoustic technician will perform digital acoustic testing of the intervention ICU bedspace environment. Photometric measures of the lighting environment will be collected over a 48-h period. Measurements will include indoor vertical and horizontal illuminance (photopic and melanopic), luminance, and reflectance values at both room and patient level. Measures will be collected with unobtrusive, validated, and calibrated instruments, including wearable devices to evaluate the lighting quality and quantity as experienced by patients.

Continuous data, such as sound levels, light levels, temperature, and humidity, will be described using mean and standard deviation and compared to previously collected bedspace data using a one-sample *t*-test. Daytime and night-time data will be evaluated and compared. The number of alarms will be described using their frequency and compared to previously collected data using chi-squared tests.

#### Component 2: stakeholder perspectives: patients, relatives, and staff

This component of the study will use qualitative methods to describe how the redesigned ICU bedspaces influence the patient experience in ICU from the perspective of patients, family members, and staff. Patients admitted to the intervention bedspaces who survive their ICU admission will be invited to take part. Data will be collected in semi-structured interviews with individual patients, accompanied by family members when available and willing to take part. Interviews will be conducted within a week after discharge from ICU but before hospital discharge. Staff from medical, nursing, allied health disciplines, and non-clinical/operational staff providing a range of services in the ICU will be invited to participate in focus groups or individual interviews. The interviewer will use a topic guide flexibly to explore participants’ experiences in the intervention bedspaces. Based on our previously conducted baseline studies [4, 31], we anticipate that between 10–15 patients and 25–30 staff will be required to reach saturation and address research questions.

The approach to analysis of the qualitative interviews has been described in detail elsewhere [4, 31]. In brief, recorded interviews will be transcribed and analysed using a framework approach [35]. Framework analysis consists of five steps: (1) familiarisation, (2) identifying thematic framework, (3) indexing, (4) charting, and (5) mapping and interpretation.

#### Component 3: sleep quality and quantity

The quality and quantity of participants’ sleep will be measured objectively, using a combination of single forehead sensor electroencephalogram (EEG) and polysomnography, as well as subjectively using validated sleep questionnaires. Sleep studies will only be commenced when the effects of relevant medications are no longer likely to impact on the quality of data collected; therefore, sleep will not be measured on participants that are deeply or moderately sedated (RASS score − 3, − 4, or − 5), or have had a general anaesthetic, drug overdose, or alcohol intoxication in the preceding 24 h. Ventilated and non-ventilated participants will be included.

Sleep will be measured continuously for a 2–4-day study period (dependent on duration of ICU admission) following recruitment using the Somfit single forehead sensor EEG (Compumedics®). This is a recently developed wearable device that is light and comfortable for patients to wear while enabling collection of high-quality EEG signals.

Sleep will be concurrently measured using a gold-standard portable polysomnography (PSG) recorder for a 24-h period. Polysomnography involves the application of sensors and electrodes for the continuous monitoring of physiological variables during sleep. The portable PSG is a small device that is worn as a belt across the patient’s thorax. Ten sensors are attached to the patient: 2 under the chin, 1 next to each eye, and 6 on the scalp. The sensors will measure and monitor muscle tone changes using electromyogram (EMG), eye movements using electrooculogram, and electrical activity in the brain using EEG. The equipment also has an inbuilt oximeter to measure pulse oximetry and can also measure patient body position as well as background light and sound. The equipment also collects electrocardiogram, leg EMG, nasal pressure/thermistor, and ribcage/abdominal movements.

Participants will be asked to complete the validated sleep in the ICU questionnaire and the Morningness-Eveningness Questionnaire before discharge from ICU [36, 37]. The aim of these questionnaires is to establish the patients’ reported quality and quantity of sleep as well as the reasons for sleep disruptions.

The data will be analysed and interpreted by expert sleep scientists. Objective measures of sleep will be described for a minimum 24-h period, including total sleep time, time awake, sleep staging (stage 1 and 2, slow wave sleep, and rapid eye movement (REM) sleep), arousal index, number of awakenings across the 24-h period, and percentage of sleep at night-time versus daytime hours. Objective sleep quality will be compared between control and interventional beds.

#### Component 4: circadian rhythms

For this component, data will be collected from environmental sensors, study records, participants, medical records, and biological samples. The following measured variables will be analysed to evaluate the circadian synchronisation of the patients and study their influence on the outcome of the patients:

- Routinely collected physiological data, including body temperature, heart rate and heart rate variability, blood pressure, pulse oximetry, and enteral/parenteral feeding rhythm
- Relevant medications with the potential to modify patients’ heart rate and blood pressure
- Quality and quantity of sleep (as described for component 3 above)
- Four-hourly blood samples for 48 h, specifically looking at cortisol, melatonin, insulin-like growth factor 1, inflammatory markers (full blood count and c-reactive protein), haemoglobin, proteomics analysis, and expression of circadian clock genes and untargeted RNA-sequencing in white blood cells

Biological measured variables will be assessed using a modified version of dryR, a statistical framework to assess differential rhythmicity [38]. The analysis is based on multiple mixed linear regression with a subsequent model selection approach based on the Bayesian information criterion to assess differential rhythmicity of the measured variables, comparing patients admitted to the intervention versus control beds. The mixed linear models will include fixed effects from a harmonic regression model and a random effect (patient–specific) on the intercept that deals with the subject–to–subject variation and dependency of the repeated measures. Rhythmic parameters including amplitude and acrophase will be computed from the selected model for each of the conditions. This will identify circadian/diurnal variables that are impacted by the intervention.

#### Component 5: delirium prevalence

The prevalence of delirium will be assessed by the completion of the confusion assessment method for the intensive care unit (CAM-ICU) twice per day (morning and afternoon) for all enrolled patients by a member of the research team.

Delirium data analysis will involve:

1. The prevalence and incidence of delirium will be calculated and compared between intervention and control bedspaces.
2. Comparison of cases and non-cases on demographic and diagnostic data and length of stay will be undertaken using methods appropriate to type of data: T-tests, chi square, and ANOVA.

#### Component 6: medium-term outcomes after ICU admission

Data on patient outcomes after ICU discharge will be collected from participants via a battery of validated self-report tools 6 months after ICU discharge. The method used for this is summarised in Table 2.

**Table 2:** Questionnaire summary

Demographics and background information	Information about baseline health status and comorbidities and return to normal occupational function and leisure activities
PROMIS applied cognition-abilities scale (PROMIS)—Short Form 8a	An 8-item measure of cognition measuring the participant’s cognitive function during the previous 7 days A total score ranging between 8 and 40 is converted to a *T*-score with 50 considered average for people slightly more unwell than the general population [39]
Hospital anxiety and depression scale (HADS)	A 14-item measure of anxiety and depression Each subscale consists of 7 items scored from 0 to 3, resulting in a score ranging between 0 and 21. A score > 7 suggests clinically significant problems [40]
The PTSD checklist for DSM-5 (PCL-5)	A 20-item screening tool that assesses the 20 DSM-5 symptoms of PTSD. A score of 31 or more is indicative of PTSD
EQ-5D-5L	A 5-item questionnaire used to measure health-related quality of life. The domains assessed are mobility, self-care, usual activities, pain/discomfort, and anxiety/depression [41] Participants will also complete retrospective EQ-5D-5L describing their baseline physical function and quality of life prior to admission and on hospital discharge
The patient employment information questionnaire	This was modified from an epilepsy study and reworded to relate to the patient’s ICU admission. It asks 7 questions around changes to employment and income since ICU admission
Ongoing sleep quality and quantity	Home sleep testing will be completed to evaluate the quality and quantity of sleep at this timepoint and correlate this with the sleep quality/quantity during ICU admission. The patient will be asked to complete the validated Pittsburgh sleep quality index (PSQI)—a 19-item self-report questionnaire that assesses sleep quality over a 1-month time interval [42]. Additionally, the Morningness-Eveningness Questionnaire will be repeated

For this study, a case of PICS will be defined as any participant with questionnaire scores outside of normal ranges indicating impaired physical (EQ-5D-5L), psychological (HADS or PCL-5), and/or cognitive function (PROMIS). The incidence of PICS will be described using proportions and associated 95% confidence intervals and compared between sub-populations, such as ICU stay < 48 h, planned vs. emergent admission, ventilated vs. non-ventilated, and type of bedspace admitted to. Multivariable regression analysis will be used to identify factors associated with PICS, or not getting PICS. The overall characteristics of the sample will be described using summary statistics. This will help inform the generalisability of our results. The number of patients approached and consented will be tabulated, together with the number who were lost to follow-up, withdrew from the study, or died.

#### Component 7: health economic evaluation

A within-trial cost-effectiveness analysis will be conducted to estimate the changes to total costs and patient outcomes associated with the intervention. The change to total costs will reflect the cost of implementing the intervention under different scenarios, offset by the economic value of ICU bed days saved from reduced ICU length of stay [43]. Planned scenarios will consider different definitions of implementation costs per intervention bedspace, which will impact the change in total costs per unit of health benefit. Definitions will reflect the cost of maintaining a bedspace that already exists in the ICU (base case), the cost of adding a new bedspace within an existing ICU, and the cost of building a new ICU to accommodate the intervention. These costs will be measured from the health system perspective. We will further consider the economic value of lost productivity from the patient perspective, which will be collected using the patient employment information questionnaire. Patient outcomes will be measured in quality-adjusted life years, which reflect patient life expectancy adjusted for health-related quality of life. Life years will be estimated using Australian life tables based on patients’ sex and age at the time of ICU admission. Quality of life, measured under component 6 via the EQ-5D-5L, will be used to estimate health utility values needed to convert life years to quality-adjusted life years.

Cost-effectiveness outcomes will be reported as an incremental cost-effectiveness ratio and net monetary benefit, for different willingness to pay thresholds. The impact of model input uncertainty on cost-effectiveness outcomes will be examined by deterministic sensitivity analysis and probabilistic sensitivity analysis. Model input distributions needed to conduct sensitivity analyses will be informed by available study data, published literature, and expert opinion where appropriate. Full details of analysis methods, assumptions, and results will be transparently reported in accordance with the 2022 Consolidated Health Economic Evaluation Reporting Standards (CHEERS 2022) checklist.

## Discussion

Despite emerging evidence of the negative impact the physical and sensory ICU environment can have on patient recovery, this is an area of critical care medicine that is commonly neglected during design of ICUs and development of patient care plans. ICU care plans usually involve setting targets, which may be targets for optimal sedation levels, ventilation settings, or amount of active exercise, but other factors essential for healing and recovery are commonly neglected, such as targets for quality and quantity of sleep overnight.

Similarly, while ICU clinicians prescribe treatments personalised to patient needs, the ICU environment is inflexible and not adapted to individual needs which necessarily change as the patient’s condition changes. With a growing evidence base demonstrating the importance of environmental factors such as natural light (or light that mimic natural light), noise reduction, biophilia (views of/access to nature), music therapy, and family support, and the rapid development of technology, there is now an opportunity to ensure that ICU bedspaces are flexible and dynamic, allowing personalisation of the space and the ability to modify the environment to suit the patient’s needs at the time. The environment can be optimised for individual patients and adjusted to aid recovery and help facilitate healing.

Previous research on environmental factors commonly addresses the outcome rather than the actual problems. For instance, studies have demonstrated that ear plugs and eye masks can help improve sleep in the ICU [44, 45]. However, this is masking rather than addressing the real problem, which is the continuous increase in noise levels in ICU, reported to increase linearly by approximately 0.4 dBA per annum [19]. Current evidence on the impact of environmental change on patient outcome is limited but suggesting that it may produce positive outcomes [29, 46–48].

The ICU of the Future project is aiming to address the commonly reported environmental problems in ICU. The protocol described in this manuscript employs mixed method methodologies to evaluate the impact of a quieter ICU with improved access to natural/circadian light, views of nature, and improved patient engagement on patient experience and outcomes. At the end of the evaluation, this study will provide new information on how an improved ICU environment can impact on patient short- and medium-term recovery, with a rigorous evaluation of multiple patient outcomes, including qualitative interviews to ensure the study also collects outcomes important to patients and their families. Importantly, a health economic evaluation will be completed, to evaluate whether the extra building and technology costs associated with upgrading the bedspace environment is offset by improved efficiencies and outcomes. An investment in getting the environment right has the potential for large, long-term positive impacts on both patient outcomes and staff health and performance, therefore producing better outcomes and cost savings. Given the significant proportion of hospital budgets invested into human resources, environmental modifications that reduce burnout and staff turnover even in small ways could quickly become cost-effective.

To our knowledge, this is the first study to investigate the impact of large environmental upgrades on patient outcomes in the ICU setting. The strengths of this protocol include the rigorous and multi-dimensional evaluation of both patient outcomes and experiences. The group of consumers (former ICU patients and their families) that helped co-design the upgraded ICU bedspaces also provided feedback and essential input into how they felt the bedspaces should be evaluated and therefore the study design, ensuring we are evaluating what is important to ICU patients. Limitations include that the study is conducted at a single site with a specific local context, potentially impacting on generalisability of data. Also, as only two bedspaces have been upgraded, the small number of beds will limit the number of patients we are able to recruit to the study. A prolonged data collection period, with regular interim data analyses, has been chosen for this reason to ensure sufficient data is collected to be able to compare the intervention bedspaces with control bedspaces. Additionally, there were no perfectly matched beds for the case–control sub-study. When the doors are closed, the implementation bedspaces function like a single room; however, the doors are much wider than the other single rooms in the unit making them functionally closer to open-plan bedspaces when the doors are open. Considering all the other single rooms have windows, it was therefore decided that the bedspaces closest in function and design to the implementation bedspaces were the three chosen open-plan and windowless ones. Lastly, during the upgrade of the two bedspaces, there were multiple changes made to the design, function, and technology in the bedspaces. Should the upgraded bedspaces demonstrate improved patient outcomes, it will not be possible to determine which feature(s) of the intervention bedspaces that contribute to these improvements, or their proportion towards contribution.

### Supplementary Information

Supplementary Material 1.

## Data Availability

The datasets analysed during the current study and statistical code are available from the corresponding author on reasonable request, as is the full protocol.

## Abbreviations

CRF: Case report forms
EEG: Electroencephalogram
EMG: Electromyogram
ICU: Intensive care units
PICS: Post-intensive care syndrome
PSG: Polysomnography
RCT: Randomised controlled trial
